# Droplet Digital RT-PCR (dd RT-PCR) Detection of SARS-CoV-2 in Honey Bees and Honey Collected in Apiaries across the Campania Region

**DOI:** 10.3390/v16050729

**Published:** 2024-05-04

**Authors:** Andrea Mancusi, Yolande Thérèse Rose Proroga, Paola Maiolino, Raffaele Marrone, Claudia D’Emilio, Santa Girardi, Marica Egidio, Arianna Boni, Teresa Vicenza, Elisabetta Suffredini, Karen Power

**Affiliations:** 1 Department of Food Security Coordination, Istituto Zooprofilattico Sperimentale del Mezzogiorno, Via Salute No. 2, 80055 Portici, Italy; andrea.mancusi@izsmportici.it (A.M.); yolande.proroga@izsmportici.it (Y.T.R.P.); santa.girardi@izsmportici.it (S.G.); 2 Department of Veterinary Medicine and Animal Production, University of Naples Federico II, 80137 Naples, Italy; paola.maiolino@unina.it (P.M.); raffaele.marrone@unina.it (R.M.); claudia.demilio@unina.it (C.D.); 3 Department of Food Safety, Nutrition and Veterinary Public Health, Istituto Superiore di Sanità, Viale Regina Elena 299, 00161 Rome, Italy; arianna.boni@iss.it (A.B.); teresa.vicenza@iss.it (T.V.); elisabetta.suffredini@iss.it (E.S.); 4 Department of Biology, University of Naples Federico II, 80126 Naples, Italy; karen.power@unina.it

**Keywords:** honey bees, bioindicators, SARS-CoV-2, Droplet Digital RT-PCR

## Abstract

Coronaviruses (CoVs), a subfamily of Orthocoronavirinae, are viruses that sometimes present a zoonotic character. Severe Acute Respiratory Syndrome Coronavirus-2 (SARS-CoV-2) is responsible for the recent outbreak of COVID-19, which, since its outbreak in 2019, has caused about 774,593,066 confirmed cases and 7,028,881 deaths. Aereosols are the main route of transmission among people; however, viral droplets can contaminate surfaces and fomites as well as particulate matter (PM) in suspensions of natural and human origin. Honey bees are well known bioindicators of the presence of pollutants and PMs in the environment as they can collect a great variety of substances during their foraging activities. The aim of this study was to evaluate the possible role of honey bees as bioindicators of the prevalence SARS-CoV-2. In this regard, 91 samples of honey bees and 6 of honey were collected from different apiaries of Campania region (Southern Italy) in four time periods from September 2020 to June 2022 and were analyzed with Droplet Digital RT-PCR for SARS-CoV-2 target genes Orf1b and N. The screening revealed the presence of SARS-CoV-2 in 12/91 in honey bee samples and in 2/6 honey samples. These results suggest that honey bees could also be used as indicators of outbreaks of airborne pathogens such as SARS-CoV-2.

## 1. Introduction

Coronaviruses (CoVs) are viruses of the Coronaviridae family, belonging to the order Nidovirales [1]. They are a highly diverse group of enveloped positive-sense single-stranded RNA viruses, with a genome ranging between 26 and 32 kb in length [2,3]. The genome codes for four main structural proteins, i.e., the spike protein (S), the envelope protein (E), the membrane protein (M), and the nucleoprotein (N), whereas non-structural proteins comprise at least 16 (nsp1 to nsp16) [4]. CoVs, like all RNA viruses, are characterized by high genetic mutation and recombination rates, which result in their increased ability to adapt to changing circumstances, which can determine high ecological diversity [5,6]. They can affect humans and animals, sometimes presenting a zoonotic character. Four CoVs are considered commonly found in humans (Human Coronavirus (HCoV) NL63, 229E, OC43, and HKU1), where they cause mild cold-like respiratory symptoms by infecting the upper respiratory tract [7,8,9]. On the contrary, zoonotic CoVs like the Severe Acute Respiratory Syndrome Coronavirus (SARS-CoV) and the Middle East Respiratory Syndrome Coronavirus (MERS-CoV) can cause severe respiratory syndromes, leading in the worst cases to death [10,11,12].

The Severe Acute Respiratory Syndrome Coronavirus-2 (SARS-CoV-2) is responsible for the Coronavirus disease (COVID-19) which has caused, since its outbreak in 2019, 774,593,066 confirmed cases and 7,028,881 deaths (https://COVID19.who.int/, accessed on 4 February 2024). The SARS-CoV-2 is genetically related to SARS-CoV-1, which emerged in two separate events in 2002 and 2003 [13] in connection to wet markets selling wild live animals, such as civets, raccoon dogs, and pangolins [14]. The transmission of SARS-CoV-2 from person to person occurs mainly via aerosols, airborne droplets (<5 μm), or droplet nuclei (5–10 μm) that contain the virus [15,16,17] and that are released through coughing and sneezing [18]. The settling viral droplets can then contaminate surfaces and fomites, as well as other particles in suspension, which can subsequently become a route of infection for susceptible people, although this route is less common than inhalation [19,20,21,22]. Moreover, while large respiratory droplets tend to fall quickly on surfaces, aerosols containing droplet nuclei can spread across further distances [23,24,25,26] and they can also be deposited in other particles in suspensions of natural and human origin that are part of particulate matter (PM) [27,28,29,30,31,32].

Due to their peculiar morphology and behavior, honey bees are well known bioindicators of the presence of pollutants in the environment as they collect a large variety of substances during their foraging activities. In particular, it has been shown that when exposed to airborne particles, they can capture PM through adhesion to their body hairs [33,34]. Therefore, honey bee colonies can be used as bioindicators of environmental SARS-CoV-2 occurrence in the application of epidemiological monitoring plans [35].

The aim of this study was to collect data on the prevalence of SARS-CoV-2 by using honey bees sampled from apiaries located across the Campania region (Southern Italy) and digital RT-PCR was used for robust virus detection and quantification, strengthening the possible use of honey bees as bioindicators. Moreover, honey samples were analyzed to evaluate the possible occurrence of SARS-CoV-2 in hive products.

## 2. Materials and Methods

### 2.1. Sampling

A total of 91 samples (S1–S91) of honey bees (*Apis mellifera*) were collected from different apiaries (Table S1 Supplementary Material) across the five provinces of Campania region (Avellino—AV; Benevento—BN; Caserta—CE; Naples—NA; Salerno—SA) at four different time points: S1–S28 were collected from September to December 2020 (T1), S29–S50 from April to July 2021 (T2), S51–S69 from September 2021 to January 2022 (T3), and S70–S91 from April to June 2022 (T4) (Table 1). For each sample, a pool of approximately fifty bees was collected from the external frames of three hives (approximately fifteen honey bees per hive), euthanized, and transported in 50 mL tubes to the laboratory of Veterinary General Pathology and Anatomical Pathology of the Department of Veterinary Medicine and Animal Productions (DMVPA), University of Naples “Federico II”, where they were stored at −20 °C until further examination. Moreover, during September 2022, six honey jars (H1–H6) were collected from six different beekeepers from the same areas (Table 2) and stored at room temperature until analysis.

### 2.2. Nucleic Acids Extraction from Honey Bees

From each sample, ten honey bees were randomly picked to create a sub-sample pool and subjected to biomolecular analysis to evaluate the possible presence of SARS-CoV-2 targets. Samples were processed according to Power et al., 2021 [36]. Briefly, honey bees were cut using a sterile blade to facilitate homogenization with a tissue lysing mechanical homogenizer (Qiagen, Hilden, Germany). Chopped samples were placed in 2 mL tubes along with a grinding metal bead and were subjected to lysis by two five-minute treatments at 50 Hz, combined with two minutes in ice to avoid overheating and preserve the integrity of the nucleic acids. RNA was extracted and purified from genomic DNA using the RNeasy Plus Mini Kit (Qiagen, Hilden, Germany), according to the manufacturer’s instructions. RNAs were kept at −80 °C until transport in dried ice to the Department of Food Safety Coordination, Istituto Zooprofilattico Sperimentale del Mezzogiorno.

### 2.3. Nucleic Acid Extraction from Honey

For each sample of honey (H1–H6), total RNA was extracted from 1 mL of honey diluted 1:10 with molecular grade water and then extracted using the MiniMag semi-automated system (bioMerieux, Marcy-l’Étoile, France) and NucliSENS reagents (bioMerieux) with slight modifications from the manufacturer’s instructions. Briefly, 2 mL of lysis buffer were added to the diluted honey and, after 20 min of lysis, 100 µL of magnetic silica were added to each sample to capture nucleic acids. Washing steps were performed according to the standard protocol, and elution was carried out in 100 µL of TE buffer. PCR inhibitors were further removed using the OneStep PCR Inhibitor Removal Kit (Zymo Research, Irvine, CA, USA). A negative extraction control sample (molecular biology water) was also processed and tested in parallel with each set of extracted samples. To assess the efficiency of extraction procedure from honey samples, 10 µL of a process control virus (Mengovirus MC_0_) were added to the samples at the beginning of the extraction procedure and viral recovery was calculated as described in ISO 15216-2:2019 [37]. Also, the removal of PCR inhibitors by the extraction procedure was assessed using an external inhibition control, as previously described.

### 2.4. Droplet Digital RT-PCR (dd RT-PCR) for SARS-CoV-2 Detection

SARS-CoV-2 detection was performed using two specific targets: the Orf1b nsp14 region [38,39] and the N gene [40,41]. Droplet digital RT-PCR was performed on Bio-Rad’s QX200 system (Bio-rad, Hercules, CA, USA). The reaction mixture (20 μL of total volume) consisted of: 5 μL of One-step RT-ddPCR Advanced Kit for Probes, 2 μL of reverse-transcriptase, 1 μL of 300 mM DTT, 5 μL of samples RNA, primers and probes in the concentrations detailed in Table 3, and nuclease-free water as required. The PCR amplification was carried out on a CFX96 Deep Well instrument (Bio-Rad) with the following thermal profile: 50 °C for 60 min and 95 °C for 10 min followed by 45 cycles of 95 °C for 15 s and 60 °C for 45 s, and a final stage at 98 °C for 10 min. After thermal cycling, the 96-well plate was read in the QX200 Droplet Reader, based on positive droplets and according to the Poisson distribution.

Results were acquired using the Bio-Rad QuantaSoft software (v1.7), counting the PCR-positive or PCR-negative droplets and providing the absolute quantification of the target sequence. The quantity of each target was expressed as the number of copies per microliter of RNA sample. Positive controls consisted of a SARS-CoV-2 RNA containing the target genes (Orf1b nsp14 region and N), supplied by Bio-Rad Laboratories.

### 2.5. Molecular Characterization of Positive Samples

In samples positive for SARS-CoV-2 by ddPCR, the molecular characterization of the virus was attempted using three previously published nested RT-PCR assays (two short-nested, PCR ID 972/973 and 974/975, and one long-nested, PCR ID 979/980) targeting the spike region [38]. Briefly, reverse transcription and first PCR amplification (PCR IDs 972, 974, and 979) was carried out using 10 μL of sample RNA, the SuperScript IV One-Step RT-PCR System (Invitrogen, Carlsbad, CA, USA), and 0.4 µM of each of the primers. Thermal profile conditions were: 45 °C for 20 min, 98 °C for 2 min, 35 cycles of 98 °C for 15 s, 58 °C for 30 s, 72 °C for either 30 s (PCR IDs 972 and 974) or 1 min and 45 s (PCR ID 979), and 72 °C for 5 min. Nested amplifications (PCR IDs 973, 975, and 980) were carried out using the DreamTaq™ Hot Start Green PCR Master Mix (Thermo Fisher Scientific, Waltham, MA, USA), with 0.4 µM of each of the primers and 2 μL of the first PCR product. The amplification conditions were 95 °C for 2 min, 45 cycles of 95 °C for 30 s, 62 °C for 30 s, 72 °C for either 30 s (PCR IDs 973 and 975) or 1 min (PCR ID 980), and 72 °C for 10 min. Standard laboratory precautions were used to avoid contamination. RNA from a B.1.1.7 SARS-CoV-2 isolate (so-called Alpha variant) was used as a positive control. The PCR products were observed by gel electrophoresis (1.5% agarose gel), purified using the GRS PCR and Gel Band Purification Kit (GRISP, Porto, Portugal), and sequenced on both strands (Eurofins Genomics, Ebersberg, Germany). Mutation analysis was performed using the GISAID CoVsurver tool (https://www.gisaid.org/epiflu-applications/covsurver-mutations-app/, accessed on 4 February 2024). The sequences were submitted to GenBank (a.n. PP346668).

## 3. Results

The screening of honey bees collected between September 2020 and June 2022 from different apiaries located in the Campania region revealed the presence of SARS-CoV-2 RNA in 12/91 samples (13.2%), namely S30 (05/2021), S37 (05/2021), S39 (04/2021), S51 (09/2021), S54 (11/2021), S60 (10/2021), S62 (10/2021), S68 (10/2021), S70 (04/2022), S73 (05/2022), S75 (05/2022), and S88 (06/2022).

**Figure 1:**
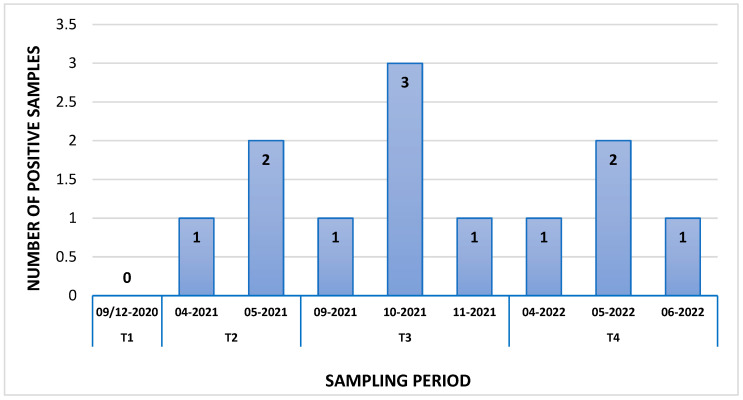
Number of positive samples for SARS-CoV-2 in the four different sampling periods (T1–T4).

In detail, the Orf1b nsp14 region of SARS-CoV-2 was detected in 12 samples, with concentrations ranging from 0.14 to 7.8 genome copies (g.c.)/µL of RNA, while the N gene was detected in 11 samples (concentration range: 0.2–1.2 g.c./µL of RNA). Overall, eleven samples displayed the presence of the virus with both PCR targets and one sample (S88) was found positive with only the Orf1b nsp14 (Table 4). Regarding the geographic distribution of SARS-CoV-2-positive samples, the virus was detected in all five provinces of the Campania region, albeit with variable detection rates: 41.7% (5/12) in the NA province, 25% (3/12) in the CE province, 17% (2/12) in the AV province, 8.3% (1/12) in the SA province, and 8.3% (1/12) in the BN province. The differences were not statistically significant (chi-square test *p* = 0.2119). SARS-CoV-2 RNA was detected in samples collected during T2 (3/12; 25%), T3 (5/12; 41.7%), and T4 (4/12; 33%), but not in T1. In detail, 1/12 samples (8.3%) were detected as positive in April 2021, 2/12 (16.6%) in May 2021, 1/12 (8.3%) in September 2021, 3/12 (25%) October 2021, 1/12 (8.3%) in November 2021, 1/12 (8.3%) in April 2022, 2/12 (16.6%) in May 2022, and 1/12 (8.3%) in June 2022 (Figure 1).

Regarding the honey samples, viral recovery ranged from 14.7% to 92.3% (average: 45.7%) and no significant PCR inhibition was detected (inhibition control amplification in samples’ reactions differing less than one Ct from the reference reaction containing molecular grade water). Two of the six honey samples (33.3%), H1 and H6 from the NA province, displayed the amplification of the Orf1b nsp14 region and of the target gene N, showing concentrations of 0.28 and 0.15 genome copies (g.c.)/µL for H1 and 0.6 and 0.23 genome copies (g.c.)/µL for H6, respectively (Table 5). For an extensive view of results, see Table S1 in the Supplementary Materials.

Amplification by nested RT-PCR ID 972/973 and the molecular characterization of the SARS-CoV-2 strain was obtained for one of the honey bee samples (S37, collected in May 2021 in the SA province), while all other attempts at amplification failed (either no amplification or unspecific amplification). Sequence analysis showed no mutations compared to the original Wuhan 2019 strain (GISAID reference sequence EPI_ISL_402124), showing that—in that specific sample—the virus present in the tested bees represented a strain preceding the massive spread in Italy of the SARS-CoV-2 Alpha variant.

## 4. Discussion

In the present study, to evaluate the possible role of honey bees as bioindicators of the prevalence SARS-CoV-2, we analyzed the samples of honey bees collected over a 2-year period (September 2020–June 2022), which almost coincided with the beginning of the COVID-19 pandemic and lasted until the point at which the pandemic was at its peak.

**Figure 2:**
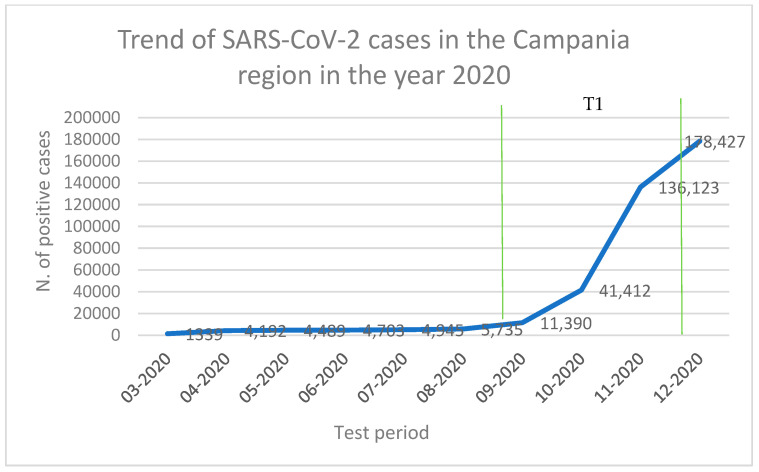
Trend of SARS-CoV-2 cases in the Campania region in the year 2020 (data obtained from ISS report).

**Figure 3:**
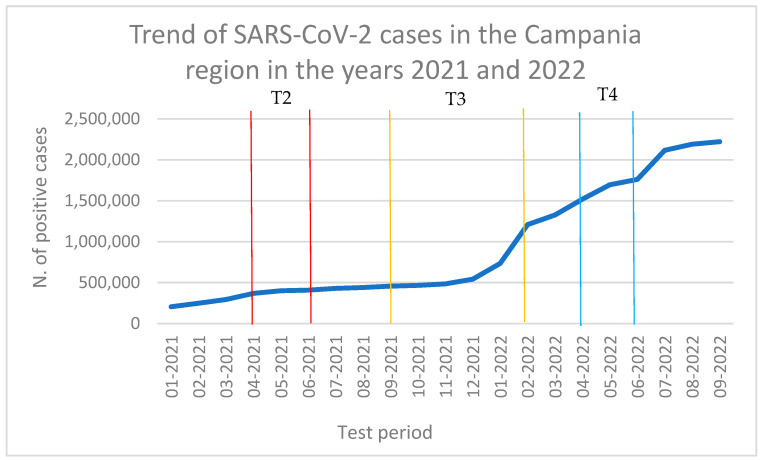
Trend of SARS-CoV-2 cases in the Campania region in the years 2021 and 2022 (data obtained from ISS report).

Our results showed that 12/91 honey bee samples were positive for SARS-CoV-2 and that no sample was positive during T1 (September 2020–December 2020), coinciding with the first winter wave of the pandemic, while 3/12 were positive during T2 (April 2021–July 2021), 5/12 during T3 (September 2021–February 2022), and 4/12 during T4 (April 2022–June 2022). These results are consistent with regional epidemiological data, since the positivity of the samples follow the increase in human cases in the population (Figure 2 and Figure 3) and the possible behavior adopted by the population. As a matter of fact, during T1, the Italian population was still obliged to use face masks and movement across the country was still partially impeded [42]. Therefore, the circulation of the virus in the air and in the PM was limited, and honey bees probably had fewer opportunities to come across the virus. On the contrary, during T2, T3, and T4, movement restrictions were loosened, face mask requirements were reduced almost everywhere [43], and the virus could more easily circulate in the air and be intercepted by honey bees. It is important to underline that none of the beekeepers or of the samplers were infected with SARS-CoV-2 at the time of sampling, nor in the month before. Since in farm animals such as buffaloes, goats, sheep, horses, rabbits, hens, pigs, or cows, SARS-CoV-2 has not been detected despite contact with their infected breeders [44], we can exclude the contamination of samples from operators’ coughing and sneezing or from the passage of fomites to honey bees, and the environmental contamination of honey bees must be assumed.

So, in accordance with other studies [35,45], our results showed that in the future, honey bees could very likely be used as indicators of outbreaks of airborne pathogens. Furthermore, by providing evidence that the molecular characterization of the pathogens detected in honey bee samples is also technically feasible, this study opens the possibility of using this approach for the identification and mapping of emerging diseases. In fact, it was observed that honey bee colonies have a higher sample capacity than an automatic environmental monitoring station, because the honey bee body has a maximum diameter of 4.5 mm [46,47], and considering that one forager may leave the hive twenty times per day flying each time for 500 m [48], it can be said that even one single honey bee can come in contact with an approximate daily volume of air of 0.16 m^3^ [49,50,51]. Assuming that in a colony there are 2000 foragers [49,50,51], those last bees may encounter 318 m^3^ of air per day, from which airborne PMs may be sampled using their hairs and brought to the colony, while an automatic station has a sampling capacity of 2.3 m^3^/h, corresponding to 55.2 m^3^ per day (UNI EN 12341:2001 and UNI EN 14907:2005).

It is worth underlining that the present study does not indicate in any way that honey bees can be infected by SARS-CoV-2. Indeed, the PCR detection of SARS-CoV-2 from the animals may be related to the virus’ attachment to pollen or other matter transported by honeybees and, given the limitations of molecular methods, may be related to a damaged or otherwise non-infectious virus. As a matter of fact, although infection with SARS-CoV-2 has been described in domestic and wild animals such as cats, dogs, minks, pet ferrets, buffaloes, otters, lions, tigers, pumas, snow leopards, gorillas, white-tailed deer, fishing cats, South American coati, spotted hyenas, Eurasian lynx, Canada lynx, hippopotami, and hamsters [52,53,54,55,56,57,58,59], it appears that insects cannot be infected with the virus. The reason probably lies in the mode of entrance of the virus into the cell, which occurs after the binding of the viral spike protein to the human angiotensin converting enzyme II (ACE2) receptor protein [60,61]. Unlike most vertebrates, which present these receptors that can tie to SARS-CoV-2 [62], only a few invertebrates have ACE2 receptors and they are very different from those of humans [63], thus preventing binding with the viral spike protein and subsequent SARS-CoV-2 infection and biological transmission [64,65].

Real-time PCR has been used since the beginning as the gold standard for the diagnosis of SARS-CoV-2 [66] and more than 150 RT-PCR COVID-19 diagnostic kits with a sensitivity of 94–100% and a specificity of 95–100% have been developed and commercialized worldwide [67]. However, this method has shown some limitations, such as long-term nucleic acid extraction, errors during sample preparation, the requirement of trained staff, and high costs for large volumes. For this reason, samples with low viral loads can be often misdiagnosed as false negative samples. Therefore, in the present study, we used a droplet digital polymerase chain reaction (dd-PCR) to overcome the limitations of RT qPCR. Droplet digital PCR is an approach based on the direct and absolute quantification of targets using the principles of dilution and partition for the reaction mix in 20,000 nanodroplets obtained with oil–water emulsion. This methodology improves the detection of targets in low concentrations and improves the accuracy of their quantification, bypassing the need for a standard curve, as is required by qPCR [68]. In fact, several studies have demonstrated the higher sensitivity and robustness of ddPCR compared to other molecular techniques, including RT-qPCR [41,68,69,70], and currently, this method is effectively used for the absolute quantification of viral load and for the analysis of gene copy number variation and circulating DNA, gene, and microRNA expression [71,72].

Regarding honey samples, data have shown that two out of six samples were positive for SARS-CoV-2. This does not mean that honey—in which the virus likely occurs as a consequence of its attachment to PM or pollen—is an element of virus transmission. It is also worth considering that the molecular detection of SARS-CoV-2 RNA is not informative in terms of the viability and infectivity of the virus; to the best of our knowledge, the latter has not been assessed in any of the studies mentioned. A realistic hypothesis is that in addition to the evident dilution effect, outdoor airborne transmission results are much less probable than the indoor route because viral integrity is strongly influenced by environmental factors, such as drying processes and sunlight exposure. The question as to whether SARS-CoV-2 is transmitted by particulate air pollution remains a controversial topic in the scientific community [34]. According to the literature, the survival of SARS-CoV-2 in the environment is substantially affected by the external conditions. An approximate loss in infectivity of 60% has been reported for different SARS-CoV-2 strains during aerosol evaporation, and the rapid inactivation of the virus on surfaces by simulated sunlight at environmental temperatures has been described. In a recent review, the half-life of SARS-CoV-2 on different inanimate surfaces at 22 °C ranged between 2 and 10 h, with a minimum of 30 min and a maximum of 5 days. Therefore, even though there seems to be a general consensus regarding the role that PM may play in severe COVID-19 due to the inflammatory effect on the respiratory system, the role of PM as a carrier of SARS-CoV-2 and as a spreader of the infection is quite controversial, as the attached virus may be non-infectious due to inactivation in the environment.

On the contrary, bibliographic studies have shown that for its antioxidant and anti-inflammatory properties, it is even used to alleviate the clinical symptoms caused by SARS-CoV-2 by reducing the elimination time of the same virus [73,74]. Furthermore, Abedi et al. (2021) evaluated the potential applications of honey and its constituent components as a possible treatment regime for COVID-19, and they have observed that the anti-inflammatory properties on proinflammatory cells and agents could lead to the prevention of SARS-CoV-2 penetration and reproduction in host cells. Additionally, they have shown that a constituent of honey called Quercetin protects the host cells by preventing the shedding of the viral coat after infiltration into the cell [75]. Finally, it was observed that honey was able to reduce the mediators of inflammation within lung infections, preventing thrombotic complications and fibrotic activity caused by COVID-19 [76].

## 5. Conclusions

In conclusion, the results of our study suggest that honey bees could very likely be used not only as bioindicators for the quality of the environment [77] but also as indicators of outbreaks of airborne pathogens, as previously indicated [35,45,78]. In fact, being accurate explorers of the ecosystem, they have the capacity to encounter a range of substances from the environment and bring them back to the hive, where they can be sampled for analysis. Clearly, honey bee-based monitoring stations cannot replace existing standard devices, as these are sensitive to physical and biological conditions that cannot be standardized. Yet, a synergy between the two methods could be promoted to obtain a greater variety of both quantitative and qualitative environmental information. Easy adaptation and handling would allow bee-based monitoring networks in virtually all inhabitable areas. For this reason, this innovative approach could be extended to other airborne pathogens that affect plants, animals, and humans and could be used to predict recurrent outbreaks like seasonal influenza. Furthermore, as previously reported [72,73,74], it is important to underline that honey samples could also be used as further indicators of virus spread in the environment without causing harm to the consumer. However, this is a preliminary study, and it is necessary to conduct further studies on different territories to ensure the reliability of the data.

## Figures and Tables

**Table 1:** Honey bees sampling was carried out over four different time periods in the Campania region.

Sample	Sampling Period	Time Period
S1–S28	September 2020–December 2020	T1
S29–S50	April 2021–July 2021	T2
S51–S69	September 2021–February 2022	T3
S70–S91	April 2022–June 2022	T4

**Table 2:** Sampling of honey carried out in September 2022.

Sample	Sampling Site
H1	Sant’Anastasia (NA)
H2	Cassano Irpino (AV)
H3	Succivo (CE)
H4	Formicola (CE)
H5	Marano (NA)
H6	Ercolano (NA)

**Table 3:** Sequence, concentrations, and reference of primers and probes used in this study.

Primer Name	Sequence	Concentrations	Reference
N_Sarbeco_F	CACATTGGCACCCGCAATC	600 nM	Corman et al., 2020 [40]
N_Sarbeco_R	GAGGAACGAGAAGAGGCTTG	800 nM	
N_Sarbeco_P	FAM-ACTTCCTCAAGGAACAACATTGCCA-BBQ *	200 nM	
2297-CoV-2-F	ACATGGCTTTGAGTTGACATCT	500 nM	La Rosa et al., 2021 [38]
2298-CoV-2-R	AGCAGTGGAAAAGCATGTGG	900 nM	
2299-CoV-2-P	FAM-CATAGACAACAGGTGCGCTC-MGBEQ *	250 nM	

* BBQ: blackberry quencher; MGB: minor groove binder; EQ: Eclipse quencher.

**Table 4:** SARS-CoV-2-positive honey bee samples.

Sample *	SARS-CoV-2 Concentration		Sampling Site
	Orf1b nsp14 g.c./µL RNA	N Gene g.c./µL RNA	
S30	1.9	0.4	Pozzuoli (NA)
S37 *	0.29	0.21	Cava dei Tirreni (SA)
S39	0.14	0.54	Teano (CE)
S51	6.1	1.2	Ercolano (NA)
S54	0.9	0.25	San Giorgio la Molara (BN)
S60	7.8	1.6	Napoli (NA)
S62	7.8	1.2	Torchiati (AV)
S68	0.7	0.3	Bellona (CE)
S70	1.7	0.22	Formicola (CE)
S73	0.23	0.2	Sant’Anastasia (NA)
S75	1.2	0.31	Marano (NA)
S88	1.0	-	Solofra (AV)

* Sample amplified by conventional nested RT-PCR and sequenced.

**Table 5:** SARS-CoV-2-positive honey samples.

Sample	SARS-CoV-2 Concentration		Sampling Site
	Orf1b nsp14 g.c./µL RNA	N Gene g.c./µL RNA	
H1	0.28	0.15	Sant’Anastasia (NA)
H6	0.6	0.23	Ercolano (NA)

## Data Availability

Data are contained within the article and supplementary materials. Further data will be shared upon request to the authors.

## Supplementary Materials

The following supporting information can be downloaded at: https://www.mdpi.com/article/10.3390/v16050729/s1, Table S1: extensive view of results.

## References

[1] (2023). ICTV. https://ictv.global/msl.

[2] Woo P.C.Y., Huang Y., Lau S.K.P., Yuen K.Y. (2010). Coronavirus Genomics and Bioinformatics Analysis. Viruses.

[3] Weiss S.R. (2020). Forty Years with Coronaviruses. J. Exp. Med..

[4] Ravi V., Saxena S., Panda P.S. (2022). Basic Virology of SARS-CoV-2. Indian J. Med. Microbiol..

[5] Cui J., Li F., Shi Z.L. (2019). Origin and Evolution of Pathogenic Coronaviruses. Nat. Rev. Microbiol..

[6] Sanjuán R., Nebot M.R., Chirico N., Mansky L.M., Belshaw R. (2010). Viral Mutation Rates. J. Virol..

[7] Santacroce L., Charitos I.A., Carretta D.M., De Nitto E., Lovero R. (2021). The Human Coronaviruses (HCoVs) and the Molecular Mechanisms of SARS-CoV-2 Infection. J. Mol. Med..

[8] Su S., Wong G., Shi W., Liu J., Lai A.C.K., Zhou J., Liu W., Bi Y., Gao G.F. (2016). Epidemiology, Genetic Recombination, and Pathogenesis of Coronaviruses. Trends Microbiol..

[9] Tang G., Liu Z., Chen D. (2022). Human Coronaviruses: Origin, Host and Receptor. J. Clin. Virol..

[10] Meo S.A., Alhowikan A.M., Al-Khlaiwi T., Meo I.M., Halepoto D.M., Iqbal M., Usmani A.M., Hajjar W., Ahmed N. (2020). Novel Coronavirus 2019-NCoV: Prevalence, Biological and Clinical Characteristics Comparison with SARS-CoV and MERS-CoV. Eur. Rev. Med. Pharmacol. Sci..

[11] Zhang T., Wu Q., Zhang Z. (2020). Probable Pangolin Origin of SARS-CoV-2 Associated with the COVID-19 Outbreak. Curr. Biol..

[12] Chan J.F.W., Kok K.H., Zhu Z., Chu H., To K.K.W., Yuan S., Yuen K.Y. (2020). Genomic Characterization of the 2019 Novel Human-Pathogenic Coronavirus Isolated from a Patient with Atypical Pneumonia after Visiting Wuhan. Emerg. Microbes Infect..

[13] Xu R.H., He J.F., Evans M.R., Peng G.W., Field H.E., Yu D.W., Lee C.K., Luo H.M., Lin W.S., Lin P. (2004). Epidemiologic clues to SARS origin in China. Emerg. Infect. Dis..

[14] Guan Y., Zheng B.J., He Y.Q., Liu X.L., Zhuang Z.X., Cheung C.L., Luo S.W., Li P.H., Zhang L.J., Guan Y.J. (2003). Isolation and Characterization of Viruses Related to the SARS Coronavirus from Animals in Southern China. Science.

[15] Leung N.H.L., Chu D.K.W., Shiu E.Y.C., Chan K.H., McDevitt J.J., Hau B.J.P., Yen H.L., Li Y., Ip D.K.M., Peiris J.S.M. (2020). Respiratory Virus Shedding in Exhaled Breath and Efficacy of Face Masks. Nat. Med..

[16] Zhang R., Li Y., Zhang A.L., Wang Y., Molina M.J. (2020). Identifying Airborne Transmission as the Dominant Route for the Spread of COVID-19. Proc. Natl. Acad. Sci. USA.

[17] Kutter J.S., Spronken M.I., Fraaij P.L., Fouchier R.A., Herfst S. (2018). Transmission Routes of Respiratory Viruses among Humans. Curr. Opin. Virol..

[18] Tang J.W., Nicolle A.D., Klettner C.A., Pantelic J., Wang L., Suhaimi A.B., Tan A.Y.L., Ong G.W.X., Su R., Sekhar C. (2013). Airflow Dynamics of Human Jets: Sneezing and Breathing—Potential Sources of Infectious Aerosols. PLoS ONE.

[19] Onakpoya I.J., Heneghan C.J., Spencer E.A., Brassey J., Plüddemann A., Evans D.H., Conly J.M., Jefferson T. (2021). SARS-CoV-2 and the Role of Fomite Transmission: A Systematic Review. F1000Res.

[20] Port J.R., Yinda C.K., Owusu I.O., Holbrook M., Fischer R., Bushmaker T., Avanzato V.A., Schulz J.E., Martens C., van Doremalen N. (2021). SARS-CoV-2 Disease Severity and Transmission Efficiency Is Increased for Airborne Compared to Fomite Exposure in Syrian Hamsters. Nat. Commun..

[21] Kraay A.N.M., Hayashi M.A.L., Hernandez-Ceron N., Spicknall I.H., Eisenberg M.C., Meza R., Eisenberg J.N.S. (2018). Fomite-Mediated Transmission as a Sufficient Pathway: A Comparative Analysis across Three Viral Pathogens. BMC Infect. Dis..

[22] Kirubananthan L., Illuri R., Rajendran R., Chandrasekaran P.R. (2021). Mechanism and Transmission Routes of COVID-19. Environmental and Health Management of Novel Coronavirus Disease (COVID-19).

[23] Guo Z.D., Wang Z.Y., Zhang S.F., Li X., Li L., Li C., Cui Y., Fu R.B., Dong Y.Z., Chi X.Y. (2020). Aerosol and Surface Distribution of Severe Acute Respiratory Syndrome Coronavirus 2 in Hospital Wards, Wuhan, China, 2020. Emerg. Infect. Dis..

[24] Fernstrom A., Goldblatt M. (2013). Aerobiology and Its Role in the Transmission of Infectious Diseases. J. Pathog..

[25] Zhou L., Ayeh S.K., Chidambaram V., Karakousis P.C. (2021). Modes of Transmission of SARS-CoV-2 and Evidence for Preventive Behavioral Interventions. BMC Infect. Dis..

[26] Greenhalgh T., Jimenez J.L., Prather K.A., Tufekci Z., Fisman D., Schooley R. (2021). Ten Scientific Reasons in Support of Airborne Transmission of SARS-CoV-2. Lancet.

[27] Sedlmaier N., Hoppenheidt K., Krist H., Lehmann S., Lang H., Büttner M. (2009). Generation of Avian Influenza Virus (AIV) Contaminated Fecal Fine Particulate Matter (PM2.5): Genome and Infectivity Detection and Calculation of Immission. Veter Microbiol..

[28] Setti L., Passarini F., De Gennaro G., Barbieri P., Perrone M.G., Borelli M., Palmisani J., Di Gilio A., Torboli V., Fontana F. (2020). SARS-Cov-2RNA Found on Particulate Matter of Bergamo in Northern Italy: First Evidence. Environ. Res..

[29] Comunian S., Dongo D., Milani C., Palestini P. (2020). Air Pollution and COVID-19: The Role of Particulate Matter in the Spread and Increase of COVID-19’s Morbidity and Mortality. Int. J. Environ. Res. Public Health.

[30] Maleki M., Anvari E., Hopke P.K., Noorimotlagh Z., Mirzaee S.A. (2021). An Updated Systematic Review on the Association between Atmospheric Particulate Matter Pollution and Prevalence of SARS-CoV-2. Environ. Res..

[31] Santurtún A., Colom M.L., Fdez-Arroyabe P., del Real Á., Fernández-Olmo I., Zarrabeitia M.T. (2022). Exposure to Particulate Matter: Direct and Indirect Role in the COVID-19 Pandemic. Environ. Res..

[32] Nor N.S.M., Yip C.W., Ibrahim N., Jaafar M.H., Rashid Z.Z., Mustafa N., Hamid H.H.A., Chandru K., Latif M.T., Saw P.E. (2021). Particulate Matter (PM2.5) as a Potential SARS-CoV-2 Carrier. Sci. Rep..

[33] Negri I., Mavris C., Di Prisco G., Caprio E., Pellecchia M. (2015). Honey Bees (*Apis Mellifera*, L.) as Active Samplers of Airborne Particulate Matter. PLoS ONE.

[34] Papa G., Capitani G., Capri E., Pellecchia M., Negri I. (2021). Vehicle-Derived Ultrafine Particulate Contaminating Bees and Bee Products. Sci. Total Environ..

[35] Cilia G., Bortolotti L., Albertazzi S., Ghini S., Nanetti A. (2022). Honey Bee (*Apis Mellifera* L.) Colonies as Bioindicators of Environmental SARS-CoV-2 Occurrence. Sci. Total Environ..

[36] Power K., Martano M., Altamura G., Piscopo N., Maiolino P. (2021). Histopathological Features of Symptomatic and Asymptomatic Honeybees Naturally Infected by Deformed Wing Virus. Pathogens.

[37] (2019). International Organization for Standardization: ISO 15216-2:2019 Microbiology of the Food Chain—Horizontal Method for Determination of Hepatitis A Virus and Norovirus Using Real-Time RT-PCR—Part 2: Method for Detection.

[38] La Rosa G., Mancini P., Bonanno Ferraro G., Veneri C., Iaconelli M., Bonadonna L., Lucentini L., Suffredini E. (2021). SARS-CoV-2 Has Been Circulating in Northern Italy since December 2019: Evidence from Environmental Monitoring. Sci. Total Environ..

[39] Pierri B., Mancusi A., Proroga Y.T.R., Capuano F., Cerino P., Girardi S., Vassallo L., Lo Conte G., Tafuro M., Cuomo M.C. (2022). SARS-CoV-2 Detection in Nasopharyngeal Swabs: Performance Characteristics of a Real-Time RT-QPCR and a Droplet Digital RT-PCR Assay Based on the Exonuclease Region (ORF1b, Nsp 14). J. Virol. Methods.

[40] Corman V.M., Landt O., Kaiser M., Molenkamp R., Meijer A., Chu D.K.W., Bleicker T., Brünink S., Schneider J., Schmidt M.L. (2020). Detection of 2019 Novel Coronavirus (2019-NCoV) by Real-Time RT-PCR. Eurosurveillance.

[41] Mancusi A., Capuano F., Girardi S., Di Maro O., Suffredini E., Di Concilio D., Vassallo L., Cuomo M.C., Tafuro M., Signorelli D. (2022). Detection of SARS-CoV-2 RNA in Bivalve Mollusks by Droplet Digital RT-PCR (Dd RT-PCR). Int. J. Environ. Res. Public Health.

[42] ISS. https://www.epicentro.iss.it/coronavirus/2020.

[43] ISS. https://www.iss.it/coronavirus.

[44] Cerino P., Buonerba C., Brambilla G., Atripaldi L., Tafuro M., Concilio D.D., Vassallo L., Conte G.L., Cuomo M.C., Maiello I. (2021). No Detection of SARS-CoV-2 in Animals Exposed to Infected Keepers: Results of a COVID-19 Surveillance Program. Future Sci. OA.

[45] Ghini S., Girotti S., Calzolari A., Sabatini A.G., Alessandrini A., Zeri L., Porrini C. (2002). Use of honeybees (*Apis mellifera* L.) as indicators of the presence of the phytopathogenic bacteria Erwinia amylovora. Insect. Soc. Life.

[46] Carreck N.L., Andree M., Brent C.S., Cox-Foster D., Dade H.A., Ellis J.D., Hatjina F., Van Englesdorp D. (2013). Standard Methods for Apis Mellifera Anatomy and Dissection. J. Apic. Res..

[47] Sauthier R., I’Anson Price R., Grüter C. (2017). Worker Size in Honeybees and Its Relationship with Season and Foraging Distance. Apidologie.

[48] Couvillon M.J., Riddell Pearce F.C., Accleton C., Fensome K.A., Quah S.K.L., Taylor E.L., Ratnieks F.L.W. (2015). Honey Bee Foraging Distance Depends on Month and Forage Type. Apidologie.

[49] He X., Wang W., Qin Q., Zeng Z., Zhang S., Barron A.B. (2013). Assessment of Flight Activity and Homing Ability in Asian and European Honey Bee Species, Apis Cerana and Apis Mellifera, Measured with Radio Frequency Tags. Apidologie.

[50] Perry C.J., Søvik E., Myerscough M.R., Barron A.B. (2015). Rapid Behavioral Maturation Accelerates Failure of Stressed Honey Bee Colonies. Proc. Natl. Acad. Sci. USA.

[51] Rodney S., Purdy J. (2020). Dietary Requirements of Individual Nectar Foragers, and Colony-Level Pollen and Nectar Consumption: A Review to Support Pesticide Exposure Assessment for Honey Bees. Apidologie.

[52] Jo W.K., de Oliveira-Filho E.F., Rasche A., Greenwood A.D., Osterrieder K., Drexler J.F. (2021). Potential Zoonotic Sources of SARS-CoV-2 Infections. Transbound. Emerg. Dis..

[53] Decaro N., Balboni A., Bertolotti L., Martino P.A., Mazzei M., Mira F., Pagnini U. (2021). SARS-CoV-2 Infection in Dogs and Cats: Facts and Speculations. Front. Veter. Sci..

[54] Delahay R.J., de la Fuente J., Smith G.C., Sharun K., Snary E.L., Flores Girón L., Nziza J., Fooks A.R., Brookes S.M., Lean F.Z.X. (2021). Assessing the Risks of SARS-CoV-2 in Wildlife. One Health Outlook.

[55] Fenollar F., Mediannikov O., Maurin M., Devaux C., Colson P., Levasseur A., Fournier P.E., Raoult D. (2021). Mink, SARS-CoV-2, and the Human-Animal Interface. Front. Microbiol..

[56] Gortázar C., Barroso-Arévalo S., Ferreras-Colino E., Isla J., de la Fuente G., Rivera B., Domínguez L., de la Fuente J., Sánchez-Vizcaíno J.M. (2021). Natural SARS-CoV-2 Infection in Kept Ferrets, Spain. Emerg. Infect. Dis..

[57] Palmer M.V., Martins M., Falkenberg S., Buckley A., Caserta L.C., Mitchell P.K., Cassmann E.D., Rollins A., Zylich N.C., Renshaw R.W. (2021). Susceptibility of White-Tailed Deer (*Odocoileus Virginianus*) to SARS-CoV-2. J. Virol..

[58] Clayton E., Ackerley J., Aelmans M., Ali N., Ashcroft Z., Ashton C., Barker R., Budryte V., Burrows C., Cai S. (2022). Structural Bases of Zoonotic and Zooanthroponotic Transmission of SARS-CoV-2. Viruses.

[59] Michelitsch A., Wernike K., Ulrich L., Mettenleiter T.C., Beer M. (2021). SARS-CoV-2 in Animals: From Potential Hosts to Animal Models. Advances in Virus Research.

[60] Jackson C.B., Farzan M., Chen B., Choe H. (2022). Mechanisms of SARS-CoV-2 Entry into Cells. Nat. Rev. Mol. Cell Biol..

[61] Huang Y., Yang C., Xu X., Xu W., Liu S. (2020). wen Structural and Functional Properties of SARS-CoV-2 Spike Protein: Potential Antivirus Drug Development for COVID-19. Acta Pharmacol. Sin..

[62] Qiu Y., Zhao Y.B., Wang Q., Li J.Y., Zhou Z.J., Liao C.H., Ge X.Y. (2020). Predicting the Angiotensin Converting Enzyme 2 (ACE2) Utilizing Capability as the Receptor of SARS-CoV-2. Microbes Infect..

[63] John S.C., Gyles E. (2019). Cozier ORCID logo; Charlotte Harrison; R. Elwyn Isaac; K. Ravi Acharya Crystal Structures of Angiotensin-Converting Enzyme from Anopheles Gambiae in Its Native Form and with a Bound Inhibitor. Biochem. J..

[64] Dehghani R., Kassiri H. (2020). A Brief Review on the Possible Role of Houseflies and Cockroaches in the Mechanical Transmission of Coronavirus Disease 2019 (COVID-19). Arch. Clin. Infect. Dis..

[65] Xia H., Atoni E., Zhao L., Ren N., Huang D., Pei R., Chen Z., Xiong J., Nyaruaba R., Xiao S. (2020). SARS-CoV-2 Does Not Replicate in Aedes Mosquito Cells nor Present in Field-Caught Mosquitoes from Wuhan. Virol. Sin..

[66] https://www.iaea.org/newscenter/news/how-is-the-covid-19-virus-detected-using-real-time-rt-pcr.

[67] https://covid-19-diagnostics.jrc.ec.europa.eu/devices.

[68] Hindson B.J., Ness K.D., Masquelier D.A., Belgrader P., Heredia N.J., Makarewicz A.J., Bright I.J., Lucero M.Y., Hiddessen A.L., Legler T.C. (2011). High-Throughput Droplet Digital PCR System for Absolute Quantitation of DNA Copy Number. Anal. Chem..

[69] Taylor S.C., Carbonneau J., Shelton D.N., Boivin G. (2015). Optimization of Droplet Digital PCR from RNA and DNA Extracts with Direct Comparison to RT-QPCR: Clinical Implications for Quantification of Oseltamivir-Resistant Subpopulations. J. Virol. Methods.

[70] Suo T., Liu X., Feng J., Guo M., Hu W., Guo D., Ullah H., Yang Y., Zhang Q., Wang X. (2020). DdPCR: A More Accurate Tool for SARS-CoV-2 Detection in Low Viral Load Specimens. Emerg. Microbes Infect..

[71] Filetti V., Falzone L., Rapisarda V., Caltabiano R., Eleonora Graziano A.C., Ledda C., Loreto C. (2020). Modulation of MicroRNA Expression Levels after Naturally Occurring Asbestiform Fibers Exposure as a Diagnostic Biomarker of Mesothelial Neoplastic Transformation. Ecotoxicol. Environ. Saf..

[72] Whale A.S., Huggett J.F., Cowen S., Speirs V., Shaw J., Ellison S., Foy C.A., Scott D.J. (2012). Comparison of Microfluidic Digital PCR and Conventional Quantitative PCR for Measuring Copy Number Variation. Nucleic Acids Res..

[73] Dilokthornsakul W., Kosiyaporn R., Wuttipongwaragon R., Dilokthornsakul P. (2022). Potential Effects of Propolis and Honey in COVID-19 Prevention and Treatment: A Systematic Review of in Silico and Clinical Studies. J. Integr. Med..

[74] Mackin C., Dahiya D., Nigam P.S. (2023). Honey as a Natural Nutraceutical: Its Combinational Therapeutic Strategies Applicable to Blood Infections—Septicemia, HIV, SARS-CoV-2, Malaria. Pharmaceuticals.

[75] Abedi F., Ghasemi S., Farkhondeh T., Azimi-Nezhad M., Shakibaei M., Samarghandian S. (2021). Possible Potential Effects of Honey and Its Main Components Against COVID-19 Infection. Dose-Response.

[76] Iba T., Levy J.H., Connors J.M., Warkentin T.E., Thachil J., Levi M. (2020). The Unique Characteristics of COVID-19 Coagulopathy. Crit. Care.

[77] Mair K.S., Irrgeher J., Haluza D. (2023). Elucidating the Role of Honey Bees as Biomonitors in Environmental Health Research. Insects.

[78] Van Der Steen J.J.M. (2016). The Colony of the Honeybee (*Apis mellifera* L.) as a Bio-Sampler for Pollutants and Plant Pathogens. Ph.D. Thesis.

